# Predictors of virological treatment failure among adult HIV patients on first-line antiretroviral therapy in Woldia and Dessie hospitals, Northeast Ethiopia: a case-control study

**DOI:** 10.1186/s12879-019-3924-4

**Published:** 2019-04-03

**Authors:** Mohammed Ahmed, Hailu Merga, Habtemu Jarso

**Affiliations:** 1 Department of Public Health, Woldia University, Woldia, Ethiopia; 2 Department of Epidemiology, Institute of health, Jimma University, Jimma, Ethiopia

**Keywords:** Virological treatment failure, First-line, Antiretroviral treatment, Case control study, Woldia, Dessie Ethiopia

## Abstract

**Background:**

Virological treatment failure is a problem that a Human Immune Virus patient faces after starting treatment due to different factors. However, there were few studies done on the predictors of virological treatment failure among adult patients on first-line antiretroviral therapy in Ethiopia in general, and no study was done in the study area in particular. Therefore, the aim of the study was to identify predictors of virological treatment failure among adult patients on first-line antiretroviral therapy in Woldiya and Dessie Hospitals, Northeast Ethiopia.

**Method:**

Hospital based case–control study was conducted in Woldia and Dessie Hospitals from from 12 August 2016–28 February 2018 on 154 cases and 154 controls among adult patients on first-line antiretroviral treatment. All cases were included and comparable controls were selected using stratified random sampling technique. Data were collected by document review using checklists and entered into Epidata version 3.1 and analyzed by SPSS version 21.

Multivariable logistic regression analysis was done to identify the independent predictors of virological treatment failure.

**Results:**

In this study, statistically higher odds of virological failure was observed among patients who had current CD4 T-cell count of < 200 mm^3^ (AOR = 2.4, 95% CI: 1.35, 4, 18) compared withCD4 T-cell count of > 200 mm^3^, current body mass index(BMI) < 16 kg/m^2^ (AOR = 4.2, 95% CI:1.85, 9.51) compared with BMI > 18.5 kg/m^2^, BMI between 16 and 18.5 kg/m^2^ (AOR = 3.72, 95% CI: 1.75, 7.92) versus BMI > 18.5 kg/m^2^, poor adherence to antiretroviral therapy (AOR = 5.4, 95% CI: 2.95, 9.97) compared with good adherence.

**Conclusion:**

This study showed that low current CD4 T-cell count and body mass index, as well as poor adherence for ART treatment predicts virological failure. Therefore, deliberate efforts are urgently needed in HIV care through improving their nutritional status by enhancing nutritional education and support, and by strengthening enhanced adherence counseling.

## Background

The global scale-up of antiretroviral treatment (ART) under the public health approach of standardized and simplified regimens has registered significant gains, with increasing access to treatment for millions of people, and a reduction in new infections and Human Immune Virus(HIV)-associated morbidity and mortality [1]. Globally, 82% of people on treatment had suppressed viral loads.Similarly, in Eastern and Southern Africa at the end of 2016 83% were virally suppressed, contributing to 29% reduction in new HIV infections between 2010 and 2016 [2].

The primary goal of ART is to prevent HIV-associated morbidity and mortality and an effective ART can reduce viremia and transmission of HIV to sexual partners by more than 96% [3]. Therefore, Monitoring people on ART is important to ensure successful treatment, identify adherence problems and determine whether ART regimens should be switched in case of treatment failure, which can be assessed in three ways: clinically, immunologically, and virologically; whichprovides an early and more accurate indication of treatment failure [4].

The World Health Organization (WHO) and Ethiopian National guideline defines virological treatment failure as plasma viral load above 1000 copies/ml (based on two consecutive viral load measurements after 3 months with enhanced adherence support) after at least 6 month of ART treatment [4–6]. Different studies showed that virological failure is a problem that an HIV patient faces after starting treatment and the magnitude of the problem is apparent in different countries:20.8% in China [7], 16% in Swaziland [8], 24.6% in Kenya [9], 24% in Mozambique [10], 41.3% in Gabon [11], 11.9% in Rwanda [12], 11% in Uganda [13], and 10.7% in Bahirdar, Ethiopia [14].

At the individual patient level, failed ART regimen or HIV drug resistance limits treatment options, complicates succession of therapy and puts the patient at increased risk for drug toxicity [15, 16] which in turn has both human and financial consequences [6]. Mathematical modeling predicts that if levels of non-nucleoside reverse transcriptase inhibitors (NNRTI) drug resistance exceed 10% in sub-Saharan Africa, between 2016 and 2020 drug resistance is predicted to be responsible for an additional 105,000 new HIV infections, 135,000 AIDS deaths, and US$ 650 million in Antiretroviral drug costs [6]. Several studies showed that various factors were positively associated with virological treatment failure such as poor adherence to treatment [9, 10, 12, 13, 17, 18], low baseline CD4 count [8, 10, 17, 19, 20], younger age [9, 10, 12, 17], longer time on ART [17, 21], being male in gender [21], advanced WHO staging [10, 20], and lower current CD4 count [17]. On the other hand, disclosed HIV status of patients, and extra baseline weight was negatively associated with virological failure [19, 22]. However, in Ethiopia, there were few studies done on the predictors of virological treatment failure among adult patients on first-line antiretroviral therapyusing routine viral load testing as a measure of treatment failure in in general, and no study was done in the study area in particular. Identifying and intervening determinants of virological treatment failure is important to achieve high treatment success rate. The aim of this study was therefore to assess the predictors of virological treatment failure among adult patients on first-line antiretroviral therapy at Woldiya and Dessie Hospitals, Northeast Ethiopia.

## Methods

### Study design and setting

Facility based unmatched case control study conducted in Woldia General and Dessie Referral Hospitals, North East Ethiopia from 12 August 2016–28 February 2018 with data extraction period from March 13–27/2018. Both Hospitals started ART services in 2005 and serving for HIV infected patients until now. Routine viral load test was started after 12 August 2016 in both hospitals. According to the information from the records of the hospitals, between 12 August 2016 to 28 February 2018, there were 9013 adult patients on first –line ART that had two consecutive documented viral load tests result in Woldiya and Dessie hospitals. From 9013 adult patients that had two consecutive documented viral load test result in both hospitals, 154 (51 in Woldia and 103 in Dessie hospital) and 534 (236 in Woldiya and 298 in Dessie hospital) of them had viral load result of above 1000 and ≤ 1000 copies/ml, respectively. All HIV-infected patients aged 15 years and above who had taken first-line ART for at least 6 months with two consecutive documented viral load test results were the source population. All cases and selected controls who had documented viral load test results from 12 August 2016–28 February 2018 were the study population. HIV-infected patients aged 15 years and above whose plasma viral load was > 1000 copies/ml in 2 consecutive viral load measurements in a 3-month interval with enhanced adherence support after at least 6 months of starting first-line ART regimen is defined as cases (virological treatment failure) whereas HIV-infected patients aged 15 years and above whose plasma viral load was < 1000 copies/mL in 2 consecutive viral load measurements after at least 6 months of starting first-line ART regimen is defined as *controls* (without virological failure).

### Sample size estimation and sampling technique

Sample size was determined using Epi Info7 version 3.5.3, by taking age less than 35 years as predictor of virological treatment failure which gave larger sample size [17], 1:1 case to control ratio, 95% Confidence interval (CI), power of 80%, and it becomes 288 (144 cases and 144 controls). However, to improve the power of the study, all of the cases (154) and comparable controls (154) were included in the study. Stratified random sampling technique was employed to select controls as study participants. First, sampling frame was prepared based on patient MRN from recorded documents of each hospital for controls separately. Then, the total sample sizes of the controls were allocated for each hospital proportionally to the number of controls. Finally, systematic random selection was applied for the selection of allocated controls (68 from Woldiya hospital and 86 from Dessie hospital) based on respective sampling interval.

### Data collection instrument and quality control

Data were extracted by document review using a structured checklist prepared in English adapted from Ethiopian Federal Ministry of Health ART clinic intake and follow up form. Data collectors and supervisors were trained for two days about the objectives of the study, contents of tools and how to collect the data before the data collection. The data was collected by 6 ART trained nurses that work at ART clinic. Two runners were used for bringing cards from the card room. The principal investigator and the supervisors were closely monitored the whole data collection process on a daily basis. To keep the quality of the study, document review checklist was prepared based on federal ministry of health of Ethiopia standard ART intake and follow up form. Data collectors and supervisors were ART trained nurse. Training was given for data collectors and supervisors before data collection and there was close follow up of data collectors by supervisors and the principal investigator including observation of how they were extracting the recorded data. Moreover, data quality was also ensured during collection, entry.

### Data processing and analysis

Data were checked for completeness, coded and, finally it was entered into Epi Data version 3.1, cleaned and analyzed by using SPSS version 21. Descriptive statistics, including frequencies mean and percentages were used to describe demographic, clinical, and treatment-related characteristics of patients. Binary logistic regression analysis was carried out for independent variables with an outcome variable to select candidate variables for multivariable analysis. Variables with a *p*-?>value < 0.25 in bivariate analysis were included into a multivariable logistic regression analysis using backward likelihood ratio method to identify the independent predictors of virological treatment failure. The final model was assessed for goodness-of-fit using Hosmer–Lemeshow test. No evidence indicating lack of fit was found (*p*-value = 0.298). Finally, variables that had significant associations with virological treatment failure were identified based on the adjusted odd ratio (AOR) with a 95% CI and *p*-value < 0.05. And also, effect modifications among independent predictors were assessed using interaction term and no effect modification was found.

## Results

### Socio-demographic characteristics of respondents

A total number of participants included in the study were 308 (154 cases and 154 controls). At baseline, the mean age of the cases and controls were 30 years (SD: 8 years) and 31 years (SD: 9 years) respectively. About 55.2% of cases and 53.9% of the controls were females; likewise, 47.4% of cases and 52.6% of the controls were married. Similarly, 38.3% of cases and 42.9% of the controls were attained primary education. By their occupation, 33.8% of cases and 30.5% of the controls were farmers. About 69.5 and 74% of the cases and controls, respectively were, disclosed their serostatus (Table 1).

**Table 1:** Baseline socio-demographic characteristics of HIV infected patients on First-line antiretroviral treatment at Woldia and Dessie hospitals, 2018

Variables	Category	Cases: N (%)	Controls: N (%)	p-value for Chi-square test
Age (years)	≤35	119 (77.3)	111 (72.1)	0.295
	>35	35 (22.7)	43 (27.9)	
Sex	Male	69 (44.8)	71 (46.1)	0.819
	Female	85 (55.2)	83 (53.9)	
Marital status	Never married	46 (29.9)	37 (24)	0.593
	Married	73 (47.4)	81 (52.6)	
	Separated	6 (3.9)	3 (2)	
	Divorced	18 (11.7)	22 (14.3)	
	Widowed	11 (7.1)	11 (7.1)	
Educational status	No education	57 (37)	55 (35.7)	0.852
	Primary	59 (38.3)	66 (42.9)	
	Secondary	28 (18.2)	24 (15.6)	
	Tertiary	10 (6.5)	9 (5.8)	
Occupational status	Farmer	52 (33.8)	47 (30.5)	0.475
	House Wife	17 (11)	19 (12.3)	
	Student	13 (8.4)	11 (7.1)	
	Merchant	11 (7.1)	9 (5.8)	
	Employed	24 (15.6)	31 (20.1)	
	Unemployed	37 (24)	37 (24)	
HIV serostatus disclosure	Yes	107 (69.5)	114 (74)	0.376
	No	47 (30.5)	40 (26)	

### Clinical related characteristics of respondents

Among the participants, 62.3% of cases and 68.2% of controls were classified as BMI > 18.5 at baseline; 59.7% of cases and 80.5% of controls had current CD4 count of > 200. Moreover, 42.9% of cases and 39% of the controls were classified as Stage III at baseline. About 83% of cases and 82.5% of controls had HIV duration > 48 months. In this study, 18.2% of cases and 22% of the controls had history of TB before starting ART, while 2% of cases and 1.3% of the controls had history of TB after starting ART (see Table 2 below).

**Table 2:** Clinical related characteristics of HIV infected patients treated at Woldia and Dessie hospitals, 2018

Variables	Category	Cases(n=154)	Controls (n=154)	p-value for Chi-square test
		Frequency (%)	Frequency (%)	
Baseline body mass index (BMI )	<16	14 (9.1)	16 (10.4)	0.349
	16-18.5	44 (28.6)	33 (21.4)	
	>18.5	96 (62.3)	105 (68.2)	
Current BMI	<16	27 (17.5)	10 (6.5)	< 0.001
	16-18.5	37 (24)	12 (7.8)	
	>18.5	90 (58.4)	132 (85.7)	
Baseline CD4 T-cell count	≤ 200	98 (63.6)	91 (59.1)	0.413
	>200	56 (36.4)	63 (40.9)	
Current CD4 T-cell count	≤ 200	62 (40.3)	30 (19.5)	<0.001
	>200	92 (59.7)	124 (80.5)	
Base line Hgb(g/dl)	≤12	51 (33.1)	43 (27.9)	0.322
	>12	103 (66.9)	111 (72.1)	
Functional status	Working	85 (55.2)	89 (57.8)	0.295
	Ambulatory	64 (41.6)	55 (35.7)	
	Bedridden	5 (3.2)	10 (6.5)	
WHO staging at baseline	stage 1	17 (11)	27 (17.5)	0.347
	stage 2	57 (37)	50 (32.5)	
	stage 3	66 (42.9)	60 (39)	
	stage 4	14 (9.1)	17 (11)	
Treatment staging^a^	T1 stage	140 (90.9)	135 (87.7)	0.643
	T2 stage	10 (6.5)	13 (8.4)	
	T3 stage	4 (2.6)	6 (3.9)	
HIV duration^b^	<24 months	7 (4.5)	7 (4.5)	0.985
	24-48 months	19 (12.3)	20 (13)	
	>48 months	128 (83)	127 (82.5)	
TB before starting ART	Yes	28 (18.2)	34 (22)	0.394
	No	126 (81.8)	120 (78)	
TB after starting ART	Yes	3 (2)	2 (1.3)	0.652
	No	151 (98)	152 (98.7)	

^a^Clinical stage of HIV patients after starting antiretroviral treatment

^b^the time includes both before and after starting ART following first HIV positive test

### Treatment related characteristics of respondents

This study revealed that 80.5% of cases and 77.3% of the controls were on ART for >48 months. About 42.2% of cases and 12.3% of the controls had history of poor adherence for ART treatment. Moreover, 90.3% of cases and 82.5% of the controls had history of Cotrimoxazole prophylaxis therapy; 5.2% of cases and 10.4 % of the controls had history of Isonized prophylaxis therapy. At the start of ART 29.9% of cases and 37% of the controls were on 1c regimen (AZT+3TC+NVP). The study showed 29.2% of cases and 11.7% of the controls had history of regimen change/individual drug (see Table 3 below).

**Table 3:** Treatment related characteristics of HIV infected patients treated at Woldia and Dessie hospitals, 2018

Variables	Category	Cases(n=154)	Controls (n=154)	p-value for Chi-square test
		Frequency (%)	Frequency (%)	
Duration on ART	<24 months	8 (5.2)	9 (5.8)	0.781
	24-48 months	22 (14.3)	26 (16.9)	
	>48 months	124 (80.5)	119 (77.3)	
Adherence level to ART treatment	Good	89 (57.8)	135 (87.7)	<0.001
	Poor	65 (42.2)	19 (12.3)	
History of CPT	Yes	139 (90.3)	127 (82.5)	0.046
	No	15 (9.7)	27 (17.5)	
History of IPT	Yes	8 (5.2)	16 (10.4)	0.089
	No	146 (94.8)	138 (89.6)	
Regimen at the start of ART	d4t+3TC+NVP	32 (20.8)	26 (16.9)	0.931
	AZT+3TC+NVP	46 (29.9)	50 (32.5)	
	AZT+3TC+EFV	30 (19.5)	30 (19.5)	
	TDF+3TC+EFV	37 (24)	38 (24.7)	
	TDF+3TC+NVP	9 (5.8)	10 (6.5)	
First line regimen currently	AZT+3TC+NVP	53 (34.4)	58 (37.7)	0.733
	AZT+3TC+EFV	32 (20.8)	31 (20)	
	TDF+3TC+EFV	48 (31.2)	50 (32.5)	
	TDF+3TC+NVP	21 (13.6)	15 (9.8)	
Change of ART regimen or individual drug	Yes	45 (29.2)	47 (30.7)	0.803
	No	109 (70.8)	107 (69.5)	
History of treatment interruption	Yes	6 (3.9)	4 (2.6)	0.520
	No	148 (96.1)	150 (97.4)	

*CPT* Cotrimoxazole Prophylaxis therapy, *IPT* Isonized Prophylaxis therapy, *AZT* Zidovudine, *d4t* Stavudine, *TDF* Tenofovir, *3TC* Lamuvidine, *NVP* Nevirapine, *EFV* Efavirnez

### Predictors of virological treatment failure

In bivariate logistic regression analysis, factors such as Marital status, WHO staging, baseline BMI, Current BMI, current CD4 T-cell count, Adherence to ART treatment, history of Cotrimoxazole Prophylaxis therapy, history of Isonized Prophylaxis therapy were associated with virological failure at *P*-value of< 0.25.

When variables that had association with virological failure in the bivariate analysis (*P*-value < 0.25) were all included in multivariable logistic regression model using backward likelihood ratio method (LR) it was found that current BMI, adherence level to ART treatment, and Current CD4T-cell count had statistically significant association with virological failure (*p*-value < 0.05).

In this study, the odds of virological failure was 2.4 times more (AOR = 2.4, 95% CI: 1.35, 4.18) among those who had Current CD4 T-cell count of ≤200 mm^3^ compared with those who had Current CD4 T-cell count of > 200 mm^3^. The odds of virological failure was 4.2 times more (AOR = 4.2, 95% CI: 1.85, 9.51) among those who had Current BMI of < 16 kg/m^2^ compared with those who had Current BMI of > 18.5 kg/m^2^. In addition, the odds of virological failure was 5.4 times more (AOR = 5.43, 95% CI: 2.95, 9.97) among those who had poor adherence compared with those who had good adherence to antiretroviral treatment (see Table 4).

**Table 4:** Multivariable logistic regression analysis shows predictors of Virological failure among first-line ART users at Woldia and Dessie Hospitals, 2018

Variables	Category	Cases: N (%)	Controls: N (%)	COR(95 %CI)	AOR(95 %CI)	p-value
Current	≤ 200	62 (40.3)	30 (19.5)	2.79(1.67,4.65)	**2.4 (1.35, 4.18)**	0.003*
CD4 count (mm^3^)	>200	92 (59.7)	124 (80.5)	1	1	
Current BMI (kg/m^2^)	<16	27 (17.5)	10 (6.5)	3.96(1.83,8.58)	**4.2 (1.85,9.51)**	0.001*
	16-18.5	37 (24)	12 (7.8)	4.52(2.24,9.14)	**3.7(1.75,7.92)**	0.001*
	>18.5	90 (58.4)	132 (85.7)	1	1	
Adherence level to ART	Good	89 (57.8)	135 (87.7)	1	1	<0.0001*
	Poor	65 (42.2)	19 (12.3)	5.2(2.91,9.24)	**5.4 (2.95, 9.97)**	

*- shows p-value <0.05 (statistically significant association) 1- shows reference category. Hosmer - lemeshow test (p-value=0.298)

## Discussion

This study was aimed to assess predictors of virological failure among first-line ART users and showed that low current CD4 T-cell count count (≤ 200mm^3^), low current BMI (< 16 kg/m^2^ and 16–18.5 16 kg/m^2^), Poor adherence to ART were found to have increased odds of virological failure.

In this study, the odds of virological failure were 2.4 times more among those who had current CD4 count of ≤200 mm^3^ compared with those who had Current CD4 T-cell count of > 200 mm^3^. The finding is supported by studies conducted in Northwestern Uganda [22], Gonder, Ethiopia [17]. It is well known that CD4 T-cell counthas an inverse relationship with viral replication and load. As patients’ immune status becomes declined, the rate of viral replication increases compared to their immune-competent counterparts. In addition, clients with compromised immunity are more vulnerable to different opportunistic infections that sustain the vicious cycle of immunity and viral replication [3].

The odds of virological failure were 4.2 times more among those who had Current BMI of < 16 kg/m^2^ compared with those who had Current BMI of > 18.5 kg/m^2^. Likewise, the odds of virological failure were 3.7 times more among those who had Current BMI between 16 and 18.5 kg/m^2^ compared with those who had Current BMI of > 18.5 kg/m^2^. This finding is supported by a study conducted Northwestern Uganda [22]. It is evident that low BMI is correlated significantly with the decrease in CD4 count and the increase in viral load by progressing in to the advanced stage of the disease, and by exposing the patients not taking ART medication (poor adherence) [23, 24].

In addition, the odds of virological failure were 5.4 times more among those who had poor adherence compared with those who had good adherence to antiretroviral treatment. This finding is supported by studies conducted in Uganda [13], Zimbabwe [20], Rwanda [12], Kenya [9], and Gonder, in Ethiopia [17]. It is grossly apparent that poor adherence to medication reduce treatment response due to suboptimal drug concentration and by doubling the viral load or viral replication, which leads to virological failure [25, 26]. This confirms that achieving long-term good adherence is indeed Achilles’ heel of successful virologic outcomes. The limitation of this study were being record based methods of data collection, which restrict the number of variables that would be studied such as psycho social factors (Depression, stigma) and differences in quality of care and service in each hospital.

## Conclusion

The present study revealed that, the key predictors for virological failure were low current (recent) CD4 count, low current (recent) body mass index, and poor adherence to antiretroviral treatment. Therefore, deliberate efforts are urgently needed in HIV care by concerned bodies like ART case managers, adherence counselors in the hospitals on patients with low body mass index, low current CD4 count (through improving their nutritional status by enhancing nutritional education and support), and improving poor adherence to ART treatment by strengthening enhanced adherence counseling.

## Abbreviations

AIDS: Acquired Immune Deficiency Syndrome
AOR: Adjusted Odd Ratio
ART: Antiretroviral Treatment
BMI: Body Mass Index
CD4: Cluster Designated 4
CI: Confidence Interval
CPT: Cotrimoxazole Prophylaxis Therapy
HIV: Human Immune Virus
IPT: Isonized Prophylaxis Therapy
NNRTI: Non Nucleoside Reverse Transcriptase Inhibitors
PLHIV: Patient Living With Human Immune Virus
RNA: Ribonucleic Acid
UNAIDS: United Nation Programme on Acquired Immune Deficiency Syndrome
WHO: World Health Organization
